# Supplementary material for: All-at-once RNA folding with 3D motif prediction framed by evolutionary information
Source: Nat Methods. 2025 Oct 3;22(10):2094–106. doi: 10.1038/s41592-025-02833-w (PMC12510878; doi:10.1038/s41592-025-02833-w)
Supplement: Supplementary file 3 — Source code to run CaCoFold-R3D. Data and results. [file 41592_2025_2833_MOESM3_ESM.gz › supplemental_material/results/CaCoFold-R3D/CaCoFold-R3D_Rfam/RF01572_DdR4.R2R.sto.pdf]

RF01572_DdR4

5′-Y R A R U G A U G A U A A C C Y A A A U A R ● A A U Y U Y U U ● Y U G A U U C C U U U Y G A U ● Y U C Y G U U C U R G A R Y R U U G G G G G A U A C U C U G U G A Y U C U ● U A U U C A U A G U U A C U C U G A Y ●
